# Arthritis increases the risk of erectile dysfunction: Results from the NHANES 2001-2004

**DOI:** 10.3389/fendo.2024.1390691

**Published:** 2024-07-03

**Authors:** Changjin Liu, Qiming Lei, Jianwei Li, Weihui Liu

**Affiliations:** ^1^ The Second Affiliated Hospital of Fujian Medical University, Quanzhou, China; ^2^ Department of Urology, The Second Affiliated Hospital of Fujian Medical University, Quanzhou, China

**Keywords:** arthritis, erectile dysfunction, NHANES, osteoarthritis, sexual dysfunction

## Abstract

**Objective:**

This study assessed the association between erectile dysfunction (ED) and arthritis.

**Methods:**

Weighted logistic regression and subgroup analyses were used to investigate the association between arthritis incidence and ED among participants in the 2001–2004 National Health and Nutrition Examination Survey database.

**Results:**

Among the participants, 27.8% and 18.5% had a self-reported history of ED and arthritis, respectively. ED was associated with arthritis (odds ratio [OR]=4.00; 95% confidence interval [CI]: 3.20–4.99; p<0.001], which remained significant after adjustment (OR=1.42, 95% CI: 1.00–1.96; p<0.001). Stratified by type of arthritis, after full adjustment, osteoarthritis remained significant (OR=1.11; 95% CI: 1.03–1.20; p=0.017), and rheumatoid arthritis (OR=1.03, 95% CI: 0.93–1.13; p= 0.5) and other arthritis (OR=1.04, 95% CI: 0.98–1.11; p=0.2) were not significantly correlated with ED. Multiple inference analyses confirmed the robustness of the results.

**Conclusion:**

Our study showed that arthritis was strongly associated with ED. There is an urgent need to raise awareness and conduct additional research on the reasons behind this association in order to implement more scientific and rational treatment programs for patients with ED and arthritis.

## 1 Introduction

Erectile dysfunction (ED) is a common disorder with a prevalence of 46.1% in American men aged 40–70 years, 42.1%–52.5% in European men aged 40–70 years, and 47.4% in Chinese men aged 40–70 years (1). ED is not life-threatening but has an enormous impact on a person’s quality of life because satisfaction with sexuality is a strong predictor of overall life satisfaction (2). Several medical, environmental, psychological, and lifestyle factors (3, 4), including cardiovascular disease, diabetes, hyperlipidemia, hypertension, metabolic syndrome, and psychological distress, have been suggested to contribute to the development of ED.

Another common chronic disease, arthritis, is increasing in prevalence; 49% of adults in the United States are predicted to develop arthritis by 2040 (5). Several previous studies have shown that various types of arthritis, including osteoarthritis (OA) (6), rheumatoid arthritis (RA) (7), gout (8), and psoriatic arthritis (9), are associated with an increased risk of ED. This may be due to the fact that arthritis, as a chronically systemic inflammatory disease, affects a wide range of systems throughout the body, including the reproductive system (10). However, some researchers found no clear association between RA and ED after deeper exploration (11). Overall, the relationship between arthritis and ED is complex and requires intensive research.

Therefore, this study investigated the relationship between arthritis and ED using data from the National Health and Nutrition Examination Survey (NHANES) from 2001–2004.

## 2 Materials and methods

### 2.1 Study population

The data utilized in this study were procured from the National Health and Nutrition Examination Survey (NHANES), which is conducted annually by the National Center for Health Statistics (NCHS), a division of the Centers for Disease Control and Prevention, with the objective of assessing the health status and behavior of unstructured populations in the United States (12). A complex multistage probability sampling design was employed in the NHANES survey to collect data representative of the population (13). All NHANES procedures were approved by the U.S. Policy for the Protection of Human Research Subjects and were reviewed and standardized annually by the NCHS Research Ethics Review Board. All individuals who participated in the survey provided informed consent, indicating their understanding of the study’s purpose.

For the purposes of this cross-sectional analysis, datasets were selected from two NHANES survey cycles: 2001–2002 and 2003–2004, because only these two cycles had complete ED and arthritis data. A total of 21,161 individuals participated in the NHANES from 2001 to 2004; the exclusion criteria were as follows: female (n=10,860) and male patients aged less than 20 years (n=5,347), missing information on ED or arthritis (n=846), and missing covariate information (n=462). A total of 3,646 patients were ultimately included in this study. Of these, 1,012 patients had a self-reported history of ED, and 795 patients had a self-reported history of arthritis.

### 2.2 Data collection and definition

We used the NHANES self-report questionnaire to diagnose ED and arthritis. All participants were asked the ED-related question, “How would you describe your ability to develop and maintain an erection sufficient for sexual intercourse?” The answers were “never,” “sometimes,” “usually,” or “almost always or nearly always.” We categorized those who answered “never” or “sometimes” as having ED. All participants were asked two questions related to arthritis. First, they were asked, “Has any doctor or other health professional ever told you that you have arthritis?” Individuals who provided a positive response were asked the follow-up question, “What type of arthritis is it?” Participants were categorized as having “no arthritis,” “rheumatoid arthritis,” “osteoarthritis,” or “other types of arthritis.”

Using multivariate-adjusted models, we identified potential confounders that may influence the association between arthritis and ED. The covariates included age, race, body mass index, weight-adjusted-waist index (WWI) (14, 15), marital status (married or living with a partner/single), poverty-to-income ratio, education level, exercise status, insurance status, alcohol consumption (whether they consumed alcohol or not), smoking status (current/former/no), hypertension, diabetes, hypercholesterolemia, cardiovascular disease, stroke, and prostate disease.

### 2.3 Statistical methods

Complex sample weights were used to address sample inclusion selection, oversampling, and non-response bias. Overall incidences of ED and arthritis were estimated using complex sample weights. Time trends in ED and arthritis incidence were assessed after adjusting for age and race. Categorical variables were compared using the card method. Logistic regression modeling was used to adjust for covariates. Odds ratios (ORs) were calculated for 95% confidence intervals (CIs) and p values. We performed sensitivity analyses of the results using multiple interpolations, which first imputed five sets of complete datasets by calling the mouse function. Subsequently, statistical analyses, such as univariate analysis and regression models, were performed within each dataset by calling a function which sets the environment for the statistical analysis. Finally, the results obtained from each analysis were combined using the pool function (16), a method of compensating for missing data based on five replicates, and the chained equation method in the Remote Method Invocation procedure to account for the effect of missing covariates on the results (17). Subgroup analyses were performed using weighted multivariate logistic regression (Model 4) for different strata of our cohort in which the association between arthritis and ED was analyzed. All statistical tests were two-sided, with a significance threshold of p<0.05. R version 4.3.2 (http://www.R-project.org, The R Foundation) was used for statistical analysis.

## 3 Results

### 3.1 Characteristics of the study participants

**Figure 1:**
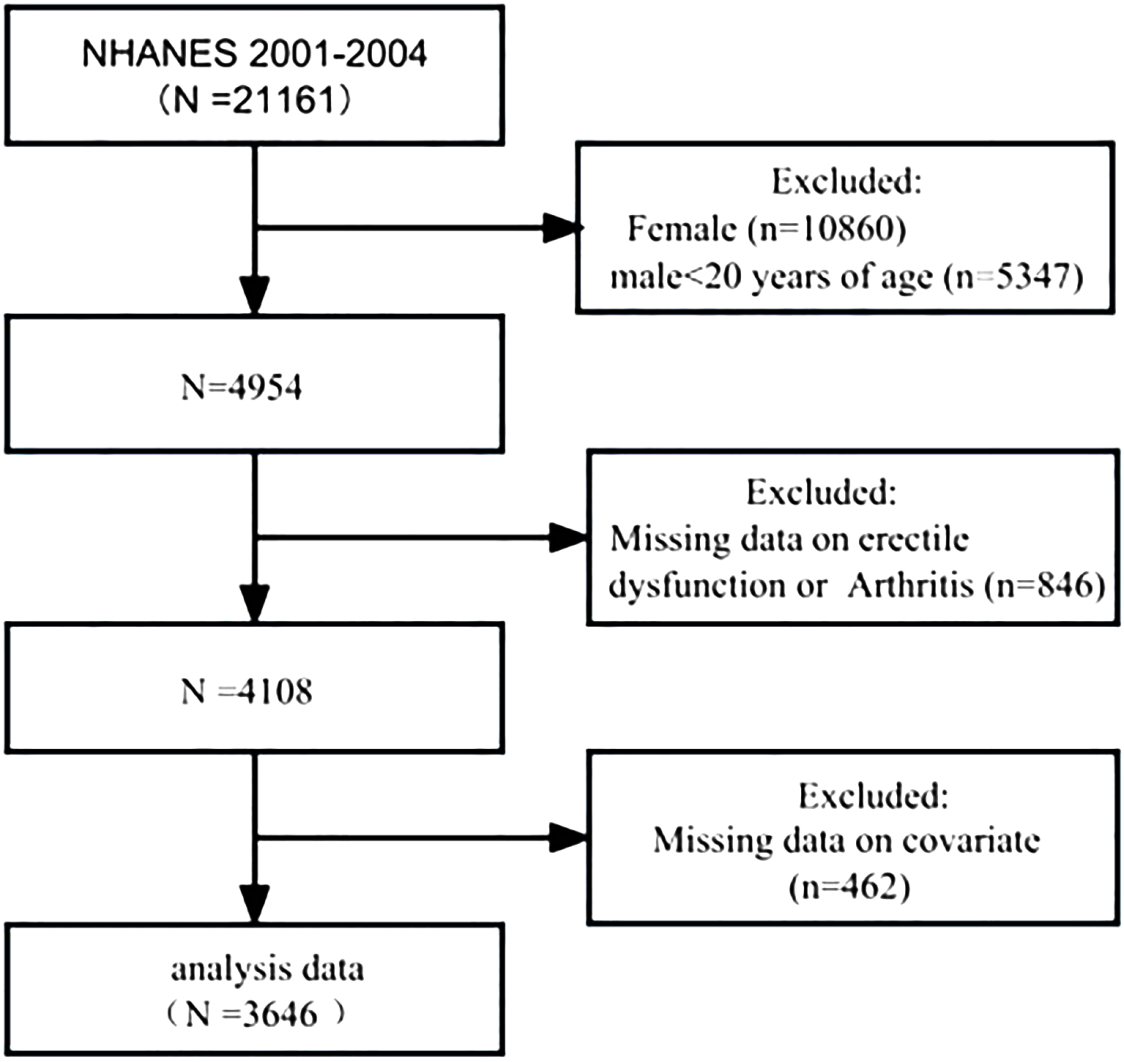
Schematic flow diagram of the inclusion and exclusion criteria for our study cohort.

Between 2001 and 2004, 21,161 participants were interviewed. Of these, 4,954 were men aged over 20 years. A total of 4,108 (83.0%) participants answered the survey questions on ED and arthritis. After adjusting for missing covariate values, 3,646 participants were included in the cohort (**Figure 1**).

Compared with participants without ED, those with ED had higher rates of all types of arthritis (p<0.001) and were more likely to be older, less educated, married or living with a sexual partner, socioeconomically disadvantaged, physically inactive, insured, not alcohol consumers, former smokers, not hypertensive, and unhealthy in terms of diabetes mellitus, dyslipidemia, obesity, cardiovascular disease, and prostate disease. No significant race differences were observed between the ED and non-ED groups (**Table 1**).

**Table 1:** Baseline characteristics of Erectile Dysfunction group versus the non-Erectile Dysfunction group.

Characteristic	Overall n= 79259578 N = 3646	Erectile Dysfunction		P Value
		No ED n= 64436827 N = 2634	ED n= 14822751 N = 1012	
Age (years)	44.9 ± 15.7	41.2 ± 13.3	61.0 ± 15.2	<0.001
Race				0.2
Mexican American	747 (7.7%)	537 (7.9%)	210 (6.8%)	
Other Hispanic	122 (4.3%)	87 (4.1%)	35 (5.1%)	
Non-Hispanic White	1,993 (74.5%)	1,394 (73.9%)	599 (77.0%)	
Non-Hispanic Black	675 (9.5%)	526 (9.8%)	149 (8.2%)	
Other Race	109 (4.0%)	90 (4.3%)	19 (2.9%)	
BMI(kg/m^2^)	28.0 ± 5.4	27.8 ± 5.2	28.9 ± 6.1	0.002
WWI	10.7 ± 0.7	10.6 ± 0.7	11.2 ± 0.7	<0.001
Diabetes				<0.001
No	3,226 (92.0%)	2,472 (95.6%)	754 (76.7%)	
Yes	372 (7.0%)	133 (3.5%)	239 (22.0%)	
Borderline	48 (1.0%)	29 (0.9%)	19 (1.3%)	
Hypertension	2,170 (65.7%)	1,797 (71.3%)	373 (41.3%)	<0.001
High cholesterol	1,344 (36.6%)	856 (32.9%)	488 (52.4%)	<0.001
Cardiovascular disease	408 (8.1%)	159 (4.6%)	249 (23.0%)	<0.001
Stroke	117 (1.8%)	33 (0.8%)	84 (6.2%)	<0.001
Prostate disease	534 (10.7%)	205 (6.7%)	329 (27.8%)	<0.001
Smoking				<0.001
Nonsmoker	1,471 (42.5%)	1,166 (45.3%)	305 (30.3%)	
Former smoker	1,190 (29.4%)	686 (25.3%)	504 (46.9%)	
Current smoker	985 (28.1%)	782 (29.4%)	203 (22.7%)	
Alcohol intake	3,029 (83.9%)	2,209 (84.5%)	820 (81.0%)	0.042
Physical Activity				<0.001
Inactive	1,361 (30.9%)	874 (28.3%)	487 (42.4%)	
Moderate	1,033 (29.2%)	684 (27.2%)	349 (37.6%)	
Vigorous	1,252 (39.9%)	1,076 (44.5%)	176 (20.0%)	
Insurance	2,887 (80.9%)	1,981 (78.7%)	906 (90.5%)	<0.001
Education level				<0.001
< High school	1,009 (16.6%)	608 (13.9%)	401 (28.4%)	
High school	894 (26.9%)	684 (27.6%)	210 (23.5%)	
> High school	1,743 (56.5%)	1,342 (58.5%)	401 (48.1%)	
Marital status				<0.001
Married or living with partner	2,523 (70.2%)	1,769 (68.6%)	754 (76.9%)	
Living alone	1,123 (29.8%)	865 (31.4%)	258 (23.1%)	
Poverty-income ratio				<0.001
<1.5	1,028 (19.0%)	706 (18.5%)	322 (21.6%)	
1.5-3.5	1,258 (32.8%)	864 (31.4%)	394 (38.8%)	
over 3.5	1,360 (48.1%)	1,064 (50.1%)	296 (39.6%)	
Arthritis				<0.001
No Arthritis	2,851 (81.5%)	2,229 (86.2%)	622 (61.1%)	
RA	164 (3.6%)	86 (2.8%)	78 (6.9%)	
OA	228 (5.2%)	103 (3.3%)	125 (13.6%)	
Others	403 (9.7%)	216 (7.6%)	187 (18.4%)	

1. Mean ± SD; n (unweighted) (%), 2. Wilcoxon rank-sum test for complex survey samples; chi-squared test with Rao & Scott’s second-order correction.

Participants with arthritis had a greater rate of having ED than did those without arthritis (p<0.001) and were older, less educated, predominantly non-Hispanic white (especially those with OA), married or living with a sexual partner, socioeconomically disadvantaged, physically inactive, insured, not alcohol consumers, ex-smokers, non-hypertensive, and had diabetes mellitus, dyslipidemia, obesity, and unhealthy in terms of cardiovascular or prostate disease. There were no significant differences in alcohol consumption between the groups with and without arthritis (**Table 2**).

**Table 2:** Baseline characteristics of arthritis group versus the non-arthritis group.

Characteristic		Arthritis				P Value
	No Arthritis n=64626120 N=2851	Total Arthritis n=14633459 N=795	RA n=2852317 N=164	OA n=4127543 N=228	Others n=7653599 N =403	
Age (years)	42.4 ± 14.8	56.1 ± 14.6	55.2 ± 13.7	60.0 ± 13.4	54.3 ± 15.1	<0.001
Race						<0.001
Mexican American	633 (8.7%)	114 (3.3%)	23 (3.4%)	20 (2.1%)	71 (4.0%)	
Other Hispanic	110 (4.9%)	12 (1.6%)	5 (4.2%)	4 (1.4%)	3 (0.8%)	
Non-Hispanic White	1,467 (72.3%)	526 (84.2%)	96 (79.5%)	174 (88.1%)	256 (83.8%)	
Non-Hispanic Black	547 (9.8%)	128 (8.0%)	37 (11.3%)	26 (5.5%)	65 (8.0%)	
Other Race	94 (4.3%)	15 (2.9%)	3 (1.6%)	4 (2.8%)	8 (3.4%)	
BMI(kg/m^2^)	27.7 ± 5.2	29.4 ± 6.2	29.0 ± 7.3	30.4 ± 6.5	29.0 ± 5.5	<0.001
WWI	10.6 ± 0.7	11.1 ± 0.7	11.1 ± 0.7	11.2 ± 0.6	11.0 ± 0.7	<0.001
Diabetes						<0.001
No	2,591 (93.9%)	635 (83.7%)	133 (83.2%)	180 (82.1%)	322 (84.8%)	
Yes	229 (5.2%)	143 (14.6%)	26 (12.4%)	42 (16.6%)	75 (14.4%)	
Borderline	31 (0.9%)	17 (1.7%)	5 (4.5%)	6 (1.3%)	6 (0.9%)	
Hypertension	1,858 (70.5%)	312 (44.4%)	64 (45.8%)	79 (37.9%)	169 (47.4%)	<0.001
High cholesterol	937 (33.0%)	407 (52.1%)	80 (48.8%)	132 (58.5%)	195 (49.8%)	<0.001
Cardiovascular disease	218 (5.4%)	190 (19.8%)	39 (21.0%)	62 (23.4%)	89 (17.4%)	<0.001
Stroke	63 (1.1%)	54 (5.1%)	15 (5.5%)	11 (3.8%)	28 (5.8%)	<0.001
Prostate disease	303 (7.8%)	231 (23.3%)	37 (16.6%)	86 (31.9%)	108 (21.2%)	<0.001
Smoking						<0.001
Nonsmoker	1,227 (45.0%)	244 (31.6%)	31 (16.0%)	69 (31.1%)	144 (37.7%)	
Former smoker	822 (26.6%)	368 (41.7%)	73 (41.2%)	123 (49.3%)	172 (37.8%)	
Current smoker	802 (28.4%)	183 (26.7%)	60 (42.8%)	36 (19.7%)	87 (24.5%)	
Alcohol intake	2,382 (84.5%)	647 (81.2%)	133 (79.4%)	189 (83.0%)	325 (80.8%)	0.071
Physical Activity						<0.001
Inactive	1,012 (28.8%)	349 (40.0%)	80 (44.5%)	86 (33.6%)	183 (41.9%)	
Moderate	743 (27.6%)	290 (36.1%)	57 (35.5%)	97 (43.2%)	136 (32.5%)	
Vigorous	1,096 (43.6%)	156 (23.9%)	27 (20.0%)	45 (23.2%)	84 (25.7%)	
Education level						<0.001
< High school	746 (15.4%)	263 (22.0%)	64 (29.0%)	56 (17.7%)	143 (21.7%)	
High school	703 (26.5%)	191 (28.6%)	41 (29.8%)	52 (26.9%)	98 (29.1%)	
> High school	1,402 (58.2%)	341 (49.4%)	59 (41.2%)	120 (55.4%)	162 (49.2%)	
Marital status						<0.001
Married or living with partner	1,921 (68.4%)	602 (78.1%)	112 (70.9%)	187 (82.4%)	303 (78.5%)	
Living alone	930 (31.6%)	193 (21.9%)	52 (29.1%)	41 (17.6%)	100 (21.5%)	
Poverty-income ratio						0.018
<1.5	786 (18.4%)	242 (21.8%)	64 (35.5%)	53 (16.9%)	125 (19.2%)	
1.5-3.5	976 (32.2%)	282 (35.3%)	56 (31.6%)	87 (38.4%)	139 (35.1%)	
over 3.5	1,089 (49.3%)	271 (42.9%)	44 (32.9%)	88 (44.7%)	139 (45.7%)	
Insurance	2,154 (78.6%)	733 (91.2%)	143 (86.2%)	213 (92.0%)	377 (92.6%)	<0.001
erectical dysfunction						<0.001
No ED	2,229 (86.0%)	405 (60.6%)	86 (64.0%)	103 (51.1%)	216 (64.4%)	
ED	622 (14.0%)	390 (39.4%)	78 (36.0%)	125 (48.9%)	187 (35.6%)	

1. Mean ± SD; n (unweighted) (%), 2. Wilcoxon rank-sum test for complex survey samples; chi-squared test with Rao & Scott’s second-order correction.

### 3.2 Association between arthritis and ED

Weighted logistic regression analysis revealed a significant positive association between arthritis and ED in all models. According to the fully adjusted model (Model 4), compared with patients without arthritis, patients with arthritis had a 42% greater risk of ED (OR=1.42; 95% CI: 1.00–1.96; p= 0.006); sensitivity analyses showed similar results in the fully adjusted model (OR=1.34; 95% CI: 1.04–1.74; p= 0.009) (**Table 3**). Among the different types of arthritis, OA was significantly associated with ED (OR = 1.11; 95% CI: 1.03–1.20; p = 0.017). Sensitivity analyses revealed similar results in the fully adjusted models (OR=1.10; 95% CI: 1.03–1.19); however, RA (OR=1.03, 95% CI: 0.93–1.13; p= 0.5) and other types of arthritis (OR=1.04; 95% CI: 0.98–1.11; p= 0.2) were not significantly associated with ED (**Table 3**).

**Table 3:** Association between Arthritis and ED.

	All arthritis and ED				RA and ED			
	Complete case		Multiple imputation		Complete case		Multiple imputation	
	OR(95% CI)	p-value	OR(95% CI)	p-value	OR(95% CI)	p-value	OR(95% CI)	p-value
Crude Model	4.00(3.20, 4.99)	***<0.001***	3.96(3.26,4.82)	***<0.001***	1.25(1.14, 1.36)	**<0.001**	1.22(1.12, 1.33)	**<0.001**
Model1	1.90 (1.42, 2.53)	***<0.001***	1.74(1.34,2.25)	***<0.001***	1.08 (1.00, 1.17)	0.06	1.05 (0.97, 1.14)	0.2
Model2	1.58 (1.16, 2.16)	***<0.001***	1.48(1.14,1.92)	***0.002***	1.04 (0.95, 1.14)	0.4	1.01 (0.93, 1.11)	0.8
Model3	1.63 (1.25, 2.12)	***<0.001***	1.51(1.18,1.93)	***<0.001***	1.06 (0.98, 1.14)	0.2	1.03 (0.95, 1.11)	0.4
Model4	1.42(1.00, 1.96)	***0.006***	1.34(1.04,1.74)	***0.009***	1.03(0.93, 1.13)	0.5	1.00(0.91, 1.10)	>0.9
	OA and ED				Other arthritis and ED			
	Complete case		Multiple imputation		Complete case		Multiple imputation	
	OR(95% CI)	p-value	OR(95% CI)	p-value	OR(95% CI)	p-value	OR(95% CI)	p-value
Crude Model	1.42(1.30, 1.54)	**<0.001**	1.41(1.31, 1.52)	**<0.001**	1.24(1.17, 1.32)	**<0.001**	1.26(1.20, 1.33)	**<0.001**
Model1	1.16 (1.08, 1.25)	**<0.001**	1.14 (1.07, 1.23)	**<0.001**	1.09(1.02, 1.15)	**0.008**	1.09(1.03, 1.15)	**0.003**
Model2	1.15 (1.06, 1.23)	**0.002**	1.13 (1.06, 1.22)	**0.002**	1.07(1.01, 1.14)	**0.035**	1.07(1.01, 1.13)	**0.018**
Model3	1.12 (1.05, 1.20)	**0.003**	1.11 (1.04, 1.18)	**0.004**	1.05(1.00, 1.11)	0.062	1.06(1.01, 1.11)	**0.031**
Model4	1.11(1.03, 1.20)	**0.017**	1.10(1.03, 1.19)	**0.017**	1.04(0.98, 1.11)	0.2	1.05(0.99, 1.11)	0.1

Crude model: no adjusted model; Model 1: adjusted for age, race; Model 2: adjusted for age, race, the family income-to-poverty ratio, education level, marital status, BMI, WWI, alcohol intake,smoking status, physical activity status, Insurance; Model 3: adjusted for age, race, cardiovascular disease, diabetes, hypertension, high cholesterol, stroke, prostate disease; Model 4: adjusted all; OR, Odds Ratio; CI, Confidence Interval.Bold and italicized values indicate p-values <0.05, which is significant.

### 3.3 Subgroup analysis of the incidence of arthritis and ED

**Figure 2:**
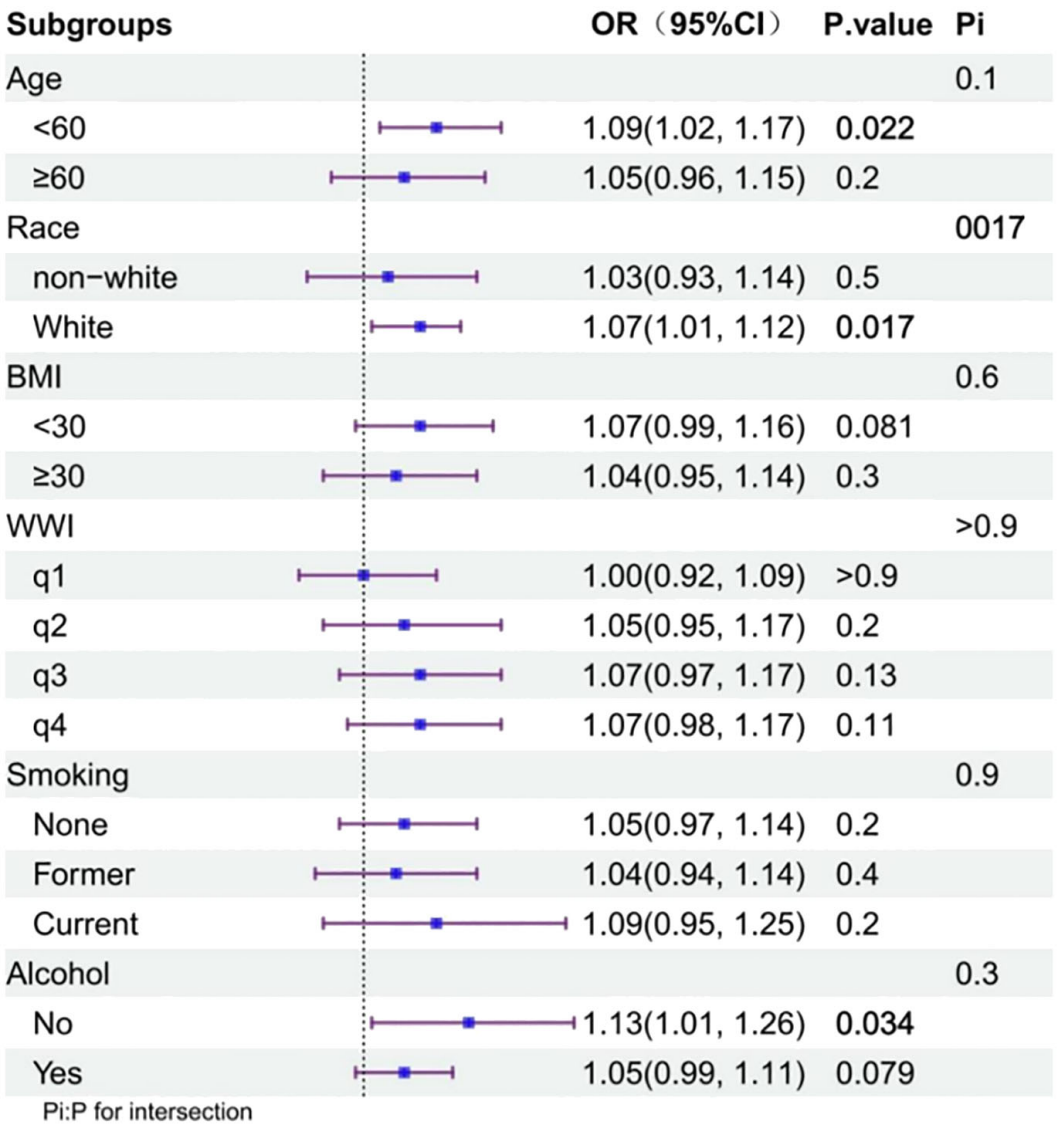
Subgroup analysis of the association between arthritis and ED status.

Subgroup analyses revealed a consistent positive correlation between the incidence of arthritis and ED across the subgroups, indicating the robustness of this correlation. Significance was maintained in the following subgroups: age <60 years, White, and no alcohol consumption (p<0.05) (**Figure 2**).

## 4 Discussion

In a pooled analysis of a nationally representative sample of 3,646 men aged >20 years in the United States, we observed a positive association between arthritis and ED incidence. This association persisted, even after adjusting for confounders. Based on the large sample size and reasonable quality control, our analysis is reliable. Previous studies have reported a higher incidence of ED in patients with inflammatory arthropathies (including RA and psoriatic arthritis) (18), ankylosing spondylitis (19), and gout (20), compared with the general population without arthritis. Similarly, in this study, 39.4% of patients with arthritis developed ED, which was much greater than the 14.0% among those without arthritis. After adjusting for confounders among the different types of arthritis, we observed that the effect of arthritis on ED incidence remained significant in patients with OA, whereas there was no significant association between RA or other types of arthritis and ED. According to our interaction analysis, White race played a role in modifying the association between arthritis and ED, with White individuals showing stronger associations, compared with other races, which may be related to the greater probability of ED disclosure in the White population (21).

According to the age-stratified analyses, there was a strong correlation between ED and the incidence of arthritis. However, the correlation between ED and arthritis found in the present study did not increase with age; rather, it was more significant in participants aged less than 60 years. Although the prevalence of arthritis in older adults is relatively high at present, it is predicted that adults aged 18–65 years will account for the majority of arthritis cases (67%) by 2040 (5); therefore, the relationship between arthritis and ED is not entirely due to aging. Moreover, previous scholars have noted that lack of exercise (22) and abnormal WWIs can lead to both arthritis and ED (14, 15). However, in the present study, after adjusting for the amount of exercise and WWI, the association between arthritis and ED remained significant. Thus, they were not the main common causes of either disorder.

The biological mechanism of inflammation may be closely related to ED in men with arthritis. It has been shown that tumor necrosis factor (TNF)-α, interleukin (IL)-1β are significantly increased in patients with OA, RA, ankylosing spondylitis, and psoriatic arthritis (18, 23). TNF-α plays a specific role in inflammation-associated ED by increasing arterial reactive oxygen species and decreasing nitric oxide levels (24), leading to deterioration of endothelium-dependent vasodilatory function in different vascular regions. Nishimatsu found that injection of the pro-inflammatory cytokine-, TNF-α-, and IL-1β-producing senescent cells in mice can impair erectile response by triggering endothelial dysfunction and nerve damage (25). Additionally, as an essential treatment for various types of arthritis, anti-TNF-α treatments play an important role in preventing the development of cavernous body dysfunction (26, 27). Zuo et al. also showed that an inflammatory state-induced decrease in endothelial nitric oxide synthase and nitric oxide levels may contribute to ED development in rats (28).

Further, Wade noted that men with moderate or severe pain had more erectile difficulties than did those without pain (29). In addition, the use of nonsteroidal anti-inflammatory drugs (NSAIDs), basic medications used in the treatment of various types of arthritis, has been shown to increase the risk of ED (30, 31). In a large clinical study, a 22% and 38% increase in the prevalence of any ED and severe ED, respectively, was observed in a group of individuals who used NSAIDs (32). Similarly, a prospective observational study in Finland (33) found a relatively increased risk of ED in men with arthritis, and men with or without arthritis that used NSAIDs had a higher risk of ED than did men who did not use NSAIDs and did not have arthritis. In addition, anti-inflammatory pain and diclofenac have been observed to inhibit the erectile process by decreasing nitric oxide levels in rats (34). Another study indicated that celecoxib might induce ED by affecting the source of reactive oxygen species, thus modulating nitric oxide breakdown (35, 36). In addition, other types of analgesics, such as opioid analgesics (37), also increase the risk of ED. Therefore, pain and analgesics may serve as a bridge between arthritis and ED.

In addition, it has been shown that the specific expression of cyclooxygenase-2 (COX-2), which plays an important role in the inflammatory microenvironment, promotes inflammatory factor-induced imbalances in cartilage proteoglycan metabolism, leading to irreversible advancement of arthritis (38). Fan et al. showed that COX-2 mRNA and protein levels were significantly higher in the synoviocytes of patients with OA and RA than in healthy tissues and were significantly higher in patients with OA than in those with RA (p < 0.05) (39). Interestingly, COX-2 protein levels are significantly elevated in the synovial fluid of patients with OA but not in those with RA (39). Moreover, it has been noted that the inflammatory state of autoimmune arthritis is associated with a bio-clock (40) that is cyclical, whereas OA usually exhibits a persistent inflammatory state. As a result, the level of inflammation in OA is higher for a longer duration than in RA, and the frequency and amount of analgesics used are increased (41). These factors may be important in the close relationship between OA and ED.

This study has several strengths. First, the survey is based on a large sample size and complex survey design, providing a comprehensive overview of the US population. Second, we adjusted for as many covariates as possible, such as general information, income, health behaviors, and comorbidities, which made our results more robust. Ultimately, the extensive data set permitted the implementation of subgroup analyses while maintaining an acceptable level of statistical power, further validating the stability of our results. However, this study has some limitations. First, because this was a cross-sectional study, causal inferences regarding arthritis and ED are not feasible. Second, the diagnoses of ED and arthritis were based on self-reported questionnaires, which may have led to misestimation of the actual number of patients with ED and arthritis. Previous studies have tested the validity of a single self-report questionnaire (42, 43). Third, our analyses were based only on the US population; therefore, the applicability of our conclusions to other populations remains to be determined. Fourth, some covariates were excluded from the analyses, such as mental illness-related cofounders (depression and anxiety), because data were not available for all participants. Finally, observational studies are susceptible to residual confounders, even after controlling for potential confounders. However, this is the first study to identify correlations between all types of arthritis and ED. We performed sensitivity analyses, and these correlations remained significant.

In conclusion, our results suggest that patients with arthritis are significantly more likely to have ED than are those without arthritis, and that arthritis is significantly associated with ED. Timely identification and treatment of ED in patients with arthritis can have a significant impact on their quality of life by avoiding the use of costly healthcare. In the future, we hope that relevant research will be conducted on populations of more races. Further research is needed to investigate the underlying molecular mechanisms to take appropriate therapeutic measures for patients with arthritis and ED.

## Data availability statement

The original contributions presented in the study are included in the article/supplementary material. Further inquiries can be directed to the corresponding authors.

## Ethics statement

The studies involving humans were approved by THE NHANES Institutional Review Board (IRB) Protocol #98-12. The studies were conducted in accordance with the local legislation and institutional requirements. The participants provided their written informed consent to participate in this study. Written informed consent was obtained from the individual(s) for the publication of any potentially identifiable images or data included in this article.

## Author contributions

CL: Data curation, Formal analysis, Methodology, Software, Validation, Visualization, Writing – original draft. WL: Conceptualization, Funding acquisition, Investigation, Project administration, Resources, Writing – review & editing. QL: Conceptualization, Data curation, Funding acquisition, Investigation, Supervision, Writing – original draft. JL: Project administration, Resources, Writing – review & editing.
